# The μ‐opioid receptor gene A118G polymorphism is associated with insecure attachment in children with disruptive mood regulation disorder and their mothers

**DOI:** 10.1002/brb3.1659

**Published:** 2020-05-18

**Authors:** Silvia Cimino, Valeria Carola, Luca Cerniglia, Silvia Bussone, Arturo Bevilacqua, Renata Tambelli

**Affiliations:** ^1^ Department of Dynamic and Clinical Sapienza—University or Rome Rome Italy; ^2^ IRCCS Santa Lucia Foundation Rome Italy; ^3^ Faculty of Psychology Psychology International Telematic University Uninettuno Rome Italy

**Keywords:** A118G, attachment style, disruptive mood regulation disorder, infant dyad, maternal care, mother, OPRM1, psychopathology

## Abstract

**Background:**

The A118G single nucleotide polymorphism (SNP) of the μ‐opioid receptor gene, with high expression of the A allele and low expression of the G allele, has been associated with emotional/behavioral dysregulation and depressive disorders and is recognized as a mediator of affiliative behavior. No study has thus far investigated this SNP in school‐age children with disruptive mood regulation disorder (DMDD). This study compared a sample of healthy children and their mothers with a sample of children with DMDD and their mothers, evaluating whether insecure attachment and psychopathological symptoms are associated with A allele‐ or G allele‐carrying mothers and children and whether caregiving capacities are associated with A allele‐ or G allele‐carrying mothers.

**Methods:**

For evaluation of their psychopathological symptoms and attachment styles, mothers filled out the CBCL/6‐18, the SCL‐90‐R, and the ECR. To evaluate the types of relationship children were experiencing with their mothers, children filled out the ECR‐revised child version and the PBI. Genotypic analyses were conducted on DNA samples obtained by buccal swabbing from children and mothers.

**Results:**

An insecure attachment style was more frequent in mothers and children carrying the G allele (G/G + A/G genotypes). In the clinical sample, G allele‐carrying children scored higher than homozygous A/A ones on the subscales of Withdrawal and Conduct Problems. G‐carrying mothers showed higher interpersonal sensitivity, depression, hostility, and paranoid ideation and provided less care than A/A mothers.

**Conclusions:**

This study offers new insights into the associations between the A118G SNP of the μ‐opioid receptor gene and emotional/behavioral functioning, attachment style in children, and psychopathology and caregiving ability in mothers.

## Graphical abstract

Insecure attachment style is more frequent in mothers and children carrying the G allele (G/G + A/G genotypes) at the μ‐opioid receptor gene.

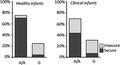

**Abbreviations**
A118G: single nucleotide polymorphism occurring in exon 1 of the μ‐opioid receptor gene
ANOVA: Analysis of Variance
CBCL/6‐18: Child Behavior Checklist/6‐18
DMDD: disruptive mood dysregulation disorder
ECR‐RC: ECR‐revised child version
ECR‐RS: Experiences in Close Relationships Scale
OPRM1: μ‐opioid receptor gene
PBI: Parental Bonding Instrument
SCL‐90/R: Symptom Checklist‐90‐Revised
SNP: single nucleotide polymorphism

**Significant outcomes**

- Insecure attachment in mothers and children appears associated with the A118G polymorphism.
- G carriers show higher psychopathological symptoms.
- G‐carrying mothers show less care toward their children than homozygous A/A mothers.

**Limitations**

- This study does not report assessment of paternal psychopathology, attachment, and caregiving.
- This study used self‐report measures to assess psychopathology, attachment, and caregiving.
- This study cannot draw causal conclusions, due to its nonlongitudinal nature.

## 1 BACKGROUND

The brain opioid system (µ, δ, and κ receptors) regulates a diverse range of physiological systems, including nociception and analgesia, respiration, gut motility, reward and euphoria, immune function, and stress responsivity (Knapman & Connor, 2015a). This system has also been described as a modulator of cognitive functions, such as emotional regulation and personality traits (e.g., impulsivity), in humans (Love, Stohler, & Zubieta, 2009; Prossin, Love, Koeppe, Zubieta, & Silk, 2010) and of motivational processes underlying reward‐related actions in preclinical models (Laurent, Leung, Maidment, & Balleine, 2012). Its pivotal role in social reward, social pain, and mood disorders is widely documented (Cinque et al., 2012; Hsu et al., 2013; Kennedy, Koeppe, Young, & Zubieta, 2006). It has been suggested that the opioid receptor system has evolutionary utility, due to its contribution to the hedonistic pleasure that can be obtained through social interaction and the pain that can derive from social exclusion (Eisenberger, 2012).

In line with these findings, the theoretical framework of the *brain opioid hypothesis of social attachment,* originally formulated by Panksepp and colleagues (Panksepp, Herman, Conner, Bishop, & Scott, 1978), postulates that a reduction in activity of the opioid system increases the desire for social companionship, while an increased activation of this system reduces the need for affiliation (Herman & Panksepp, 1978; Stein, van Honk, Ipser, Solms, & Panksepp, 2007). Consistent with this hypothesis, evidence has been collected in many animal species using several distinct behavioral parameters (Barr et al., 2008; Moles, Kieffer, & D’Amato, 2004).

Genetic variations affecting the function of µ‐opioid receptors have been shown to influence social behavior in both animals (Barr et al., 2008; Moles et al., 2004) and humans (Copeland et al., 2011). More than 100 single nucleotide polymorphisms (SNP) have been detected in the μ‐opioid receptor gene (OPRM1). A functional SNP (rs1799971) occurring in exon 1 of this gene results in the nonsynonymous substitution of a guanine (G) for an adenine (A) at nucleotide position 118 (A118G), which leads to an asparagine to aspartic acid amino acid change at position 40 (N40D), which abolishes an N‐glycosylation site. This substitution has been associated with lower mRNA transcription and translation (Zhang, Wang, Johnson, Papp, & Sadée, 2005) and with lower opioid signaling efficiency in specific brain areas (Mague et al., 2009; Oertel et al., 2012; Ray et al., 2011). Considering the inhibitory action of opioid receptors on neuronal activity, a reduced expression of the μ‐opioid receptor gene results in increased cell reactivity, with an apparent dominant effect (see, for example, Bart et al., 2006; Fillingim et al., 2005; Ray & Hutchison, 2007; Wand et al., 2011). Consequently, compared to A/A homozygotes, patients with at least one G allele experience greater pain intensity during surgery and require larger doses of opiates to relieve postsurgical pain (Sia et al., 2013; Tan et al., 2009). In addition, G allele carriers exhibit a propensity to experience more social pain (Menon et al., 2012; Tchalova, Sadikaj, Moskowitz, Zuroff, & Bartz, 2019) and increased emotional dysregulation and neural activation in response to social rejection. In contrast to A/A homozygotes, G allele carriers also exhibit behavioral retraction to angry faces, higher levels of rejection sensitivity, and high levels of fearful attachment regardless of the quality of their early maternal care (Bertoletti, Zanoni, Giorda, & Battaglia, 2012; Troisi et al., 2012; Way, Taylor, & Eisenberger, 2009), strongly suggesting that the A118G genotype has a moderating role in the effects of early maternal care on adult attachment style.

Two studies suggest that the A118G SNP is involved in cortisol secretion, with G allele carriers showing higher resting cortisol and blunted cortisol response to a psychosocial stressor (Lovallo et al., 2015; Mague & Blendy, 2010). This evidence is in line with experimental results showing a role for the A118G SNP in modulating the sensitivity to both positive and negative environmental stress factors via the reward system response and stress reactivity (Carver, Johnson, & Kim, 2015). The susceptibility of G carriers to developing psychopathological symptoms after exposure to stressful conditions has repeatedly been reported (Kreek & LaForge, 2007). Slavich, Tartter, Brennan, and Hammen (2014) have found that G‐carrying adolescents are more severely depressed and are more likely to develop a depressive disorder following major life stress than are A/A homozygotes.

Recently, a role has been recognized for the A118G SNP in mediating affiliative behavior, such as affectionate relationships and attachment (Nobile et al., 2019; Troisi et al., 2011). Importantly, it has been shown that this SNP is involved in social attachment and the social reward system in children (Copeland et al., 2011).

Although the research in this field has focused on the A118G polymorphism and on several psychopathological traits, such as stress response, social anhedonia (Lovallo et al., 2015; Troisi et al., 2011), and heightened sensitivity to social rejection (Way et al., 2009), and on psychopathologies such as addictive behaviors, depression, and suicide (Kennedy et al., 2006; Nobile et al., 2019; Schwantes‐An et al., 2016), no studies have confirmed its association with the disruptive mood dysregulation disorder (DMDD) in school‐age youths. This clinical condition has a prevalence of 2%–5% in pediatric psychiatric populations, and it has been recently included among the depressive disorders in DSM‐5 (American Psychiatric Association, 2013), being characterized by a severe impairment in the emotion and behavioral regulatory processes. Deriving from the severely hindered regulation, individuals with DMDD typically show chronic (nonepisodic) irritability and low frustration tolerance that is frequently associated with temper outbursts, disrupted relationships with family members and peers, difficulties in building and maintaining significant bonds and friendships with others, school‐related difficulties, and problems in participating in activities that are generally enjoyed by healthy children. The persistent and severe difficulties of children with DMDD in establishing and maintaining satisfactory relationships with others could result in an experience of social rejection and pain that can be amplified in G‐carrier children.

Evidence suggests that DMDD in youth is predictive of unipolar and mood disorders in adulthood (Copeland, Angold, Costello, & Egger, 2013; Leibenluft, 2011). It is therefore crucial to identify genetic features relating to this disorder.

### 1.1 Aims of the study

In agreement with the effect of the A118G SNP differentiating A/A subjects from G allele carriers (A/G and G/G), we have evaluated whether subjects enrolled in the present study, sorted for the presence or absence of the G allele, may also be differentiated regarding (a) secure/insecure attachment; (b) psychopathological symptoms; and (c) caregiving ability. Moreover, since the published literature suggests that the G allele may be more likely to be present in individuals with depressive disorders, we have also specifically investigated whether, in a sample of children with DMDD and their mothers, compared with normal controls, (a) insecure attachment is more frequent in G carriers; (b) psychopathological symptoms are higher in G carriers; and (c) caregiving capacities are lower in G‐carrying mothers.

## 2 METHODS

### 2.1 Participants

Thanks to a collaboration with mental health services and schools of Central Italy, we recruited 213 families (composed of mothers and one or more children) with children aged from 8 to 9 years, who were diagnosed for the disruptive mood dysregulation disorder and a group of nonclinical families, matched for demographic characteristics. From the total sample, we excluded 2 families of mothers who did not understand Italian, 3 families of parents who were not the biological parents of the children, 10 families with children with reported mental and/or physical disabilities, 8 families in which one or more members was following a pharmacological or psychological treatment; 13 families who did not complete all the questionnaires, and 27 families who refused to participate in the study.

The final sample was composed of 150 children, 72 females and 78 males aged from 8 to 9 years (*M* = 8.2; *SD* = 0.9), and their mothers, aged from 35.1 to 41.3 years (*M* = 38.3 years *SD* = 2.5). Eighty‐five mother–child pairs were included in the clinical group, 65 pairs in the control group. All families were Caucasian, and most of them had a middle–high socioeconomic level according to the Hollingshead's social status index (Hollingshead, 1975). A large majority (96%) of the families were intact family groups. Furthermore, 86.7% of children were first‐born for both parents. Confounding variables, such as alcohol use, smoking, drug abuse, current medical illness, traumatic experiences, and socialeconomic status, were assessed through an ad hoc anamnestic questionnaire specifically created for this study.

The recruitment in the mental health services was made possible by pre‐existent research agreements with the Authors’ institutions; a group of psychologists, specifically trained for the purposes of the study, presented the project to families at schools, after receiving the consent of the primary school headmaster. Written informed consent, explaining the scope and phases of the study, was obtained from parents. On the other hand, children were orally informed. Procedures were approved by the Ethics Committee of the Dynamic and Clinical Psychology Department of the University “Sapienza” of Rome (project number: 27/2016) and complied with institutional guidelines and the Declaration of Helsinki. Written informed consent was obtained from mothers and orally by children.

Mothers filled out the CBCL/6‐18 (Achenbach & Rescorla, 2001) to describe their children emotional/behavioral functioning, the SCL‐90/R (Derogatis, 1994) to self‐report their own psychopathological symptoms and the ECR‐RS (Fraley, Heffernan, Vicary, & Brumbaugh, 2011) to self‐report their attachment style. Children filled out the ECR‐RC (Brenning, Soenens, Braet, & Bosmans, 2011) and the PBI (Parker, Tupling, & Brown, 1979) to self‐report the quality of their attachment to their mothers and the quality of perceived caregiving. All questionnaires were compiled at home. On a different day, saliva samples were obtained from mothers and children (a group of trained psychologists administered both biological and psychological assessment; the order of administration of questionnaires and biological sample was randomly counterbalanced).

### 2.2 Procedure

#### 2.2.1 Procedure for biological sampling

Saliva samples from children and mothers were collected by buccal swabs (Isohelix Swab Pack, Cell Product Ltd, Harriestam, UK) following the manufacturer protocol; they were slightly chilled by Normative ice (+4°C) and transported to the laboratory for further processing. After buccal swabs were gathered, mothers and children independently filled out self‐report and report form questionnaires.

#### 2.2.2 DNA isolation and genotyping

After epithelial cell samples were collected from the buccal swabs by gentle centrifugation (speed and time), DNA isolation was performed using the Buccal‐Prep Plus DNA isolation kit (Isohelix, Cell Product Ltd., Harriestam, UK) following the manufacturer's instructions. DNA samples were genotyped for the A118G (rs1799971) SNP by the TaqMan® genotyping protocol. According to this protocol, 10 ng of DNA was poured into each well of the reaction plate, with 2.50 µl of TaqMan® Universal PCR Master Mix (Catalog#: 4371353; Applied Biosystems, Branchburg, NJ) and 0.25 µl of the corresponding TaqMan SNP genotyping assay (Catalog#: 4351379; Applied Biosystems), containing VIC and FAM probes. The plate was then run on an Applied Biosystems 7900HT Fast Real‐Time PCR under the following thermal cycling conditions: an initial denaturation at 95°C for 10 min followed by 40 cycles of denaturation at 92°C for 15 s and annealing at 60°C for 60 s. Finally, genotypes were assigned by registering the fluorescence emissions from each well at the corresponding VIC and FAM dye wavelengths. Genotyping of each DNA sample was performed twice in blind conditions.

### 2.3 Measures

#### 2.3.1 Assessment of psychopathological symptoms in mothers

Mothers were administered with the Symptom Checklist‐90 item Revised (SCL‐90‐R Derogatis, 1994), a 90‐item self‐report questionnaire measuring psychological symptoms and distress in adults from general and clinical populations. The SCL‐90‐R is rated on a Likert scale from 0 (not at all) to 4 (extremely) and asks participants to report whether they have suffered in the preceding week from symptoms of somatization (e.g., headaches), obsessive‐compulsivity (e.g., having to check and double‐check one's actions), interpersonal sensitivity (e.g., feeling that people are unfriendly or disliking), depression (e.g., feeling blue), anxiety scale (e.g., feeling fearful), hostility (e.g., having urges to beat, injure, or harm someone), phobic anxiety (e.g., feeling afraid to go out of the house alone), paranoid ideation (e.g., the idea that one should be punished for their sins), and psychoticism (e.g., having thoughts that are not one's own). Aside from these nine primary scales, the questionnaire provides a global severity index (GSI), which is used to determine the severity and degree of psychological distress. The SCL‐90‐R, in its Italian version (Prunas, Sarno, Preti, Madeddu, & Perugini, 2012), showed good internal coherence (α = 0.88) in this study. In this study, we used the GSI scores to assess mothers’ psychopathological risk.

#### 2.3.2 Assessment of emotional and behavioral functioning in children

Mothers also filled out the Italian version of the Child Behavior Checklist/6–18 (CBCL/6‐18 Frigerio & Montirosso, 2011), which is one of the most widely used instruments to assess child and adolescent psychopathology in both epidemiological and clinical samples. The CBCL/6–18 is a 113‐item informant‐report questionnaire that asks parents (mothers and fathers independently) to rate specific emotional/behavioral problems of their child during the preceding six months. Items are rated on a three‐point Likert scale ranging from 0 (not true) to 2 (very true or often true), and they are grouped into eight empirically based syndrome scales: Anxious/Depressed, Withdrawn/Depressed, Somatic Complaints, Social Problems, Thought Problems, Attention Problems, Rule‐Breaking Behavior, and Aggressive Behavior. In this tool, the scales Anxious/Depressed, Withdrawn/Depressed, and Somatic Complaints are grouped into the subscale of Internalizing Problems; Rule‐Breaking Behavior and Aggressive Behavior are grouped into the subscale of Externalizing Problems; in addition, Social Problems, Thought Problems, and Attention Problems (not grouped into any subscale) are also considered. For the aims of the present study, we used DSM‐5 oriented scales (Depressive Problems, Anxiety Problems, Somatic Problems, Attention Deficit/Hyperactivity Problems, Oppositional Defiant Problems, and Conduct Problems). We used the clinical cutoffs for the DSM‐oriented scales and thus regarded these variables as categorical.

#### 2.3.3 Assessment of perceived maternal care

Maternal care experienced by children was measured using the Italian version of the Parental Bonding Inventory (Scinto, Marinangeli, Kalyvoka, Daneluzzo, & Rossi., 1999). The PBI includes two subscales, one assessing maternal warmth/care and one assessing maternal overprotection. Participants report how true each statement is regarding their own experience, on a four‐point scale. The PBI has been found to have good reliability and validity, long‐term stability, satisfactory construct and convergent validity, and to be independent of mood effects (Parker et al., 1979).

#### 2.3.4 Assessment of attachment style

Attachment style in mothers was assessed through the ECR‐RS (Fraley et al., 2011). This self‐report tool is a nine‐item self‐report instrument designed to measure attachment‐related anxiety (items 1–6) and avoidance (items 7–9) in close relationships (with the mother or mother‐like figure, father or father‐like figure, romantic partner, and best friend). Subjects are instructed to respond to the questions by considering their relationship with each relational target. In the present study, we asked mothers to respond with regard to their own mothers. The scale is rated on a seven‐point Likert scale that ranges from 1 (strongly disagree) to 7 (strongly agree). The total subscale score consists of the mean of the items and ranges from 1 to 7, with higher scores indicating higher attachment avoidance or anxiety. Global measures of secure vs. insecure attachment may be obtained by considering the orthogonal dimensions of anxiety and avoidance.

Attachment style in children was measured through the ECR‐RC (Brenning et al., 2011). This self‐report tool assesses attachment anxiety (e.g., “I worry that my mother/father does not really love me”) and attachment avoidance (e.g., “I prefer not to tell my mother/father how I feel deep down”) in children and adolescents. The recently developed short form of ECR‐RC consists of 12 items (six for anxiety and six for avoidance) on a five‐point Likert scale. Scores across items are averaged to provide an anxiety and an avoidance score, respectively, with higher scores indicating a more anxious or avoidant attachment. In the present study, we used the Italian version of the questionnaire (Lionetti, Mastrotheodoros, & Palladino, 2017).

### 2.4 Statistical analyses

All data were initially checked for homogeneity of variance by Levene's test. Comparisons between groups were performed using the chi‐square test on categorical measures. All other parameters were subjected to parametric, one‐way or two‐way analysis of variance (ANOVA) followed, in cases of significance (*p* < .05), by Duncan's test. Statistical analyses were carried out by Statistica software Version 12.0 (StatSoft).

## 3 RESULTS

### 3.1 Frequency of secure versus insecure attachment in G‐carrying mothers and children

**Figure 1:**
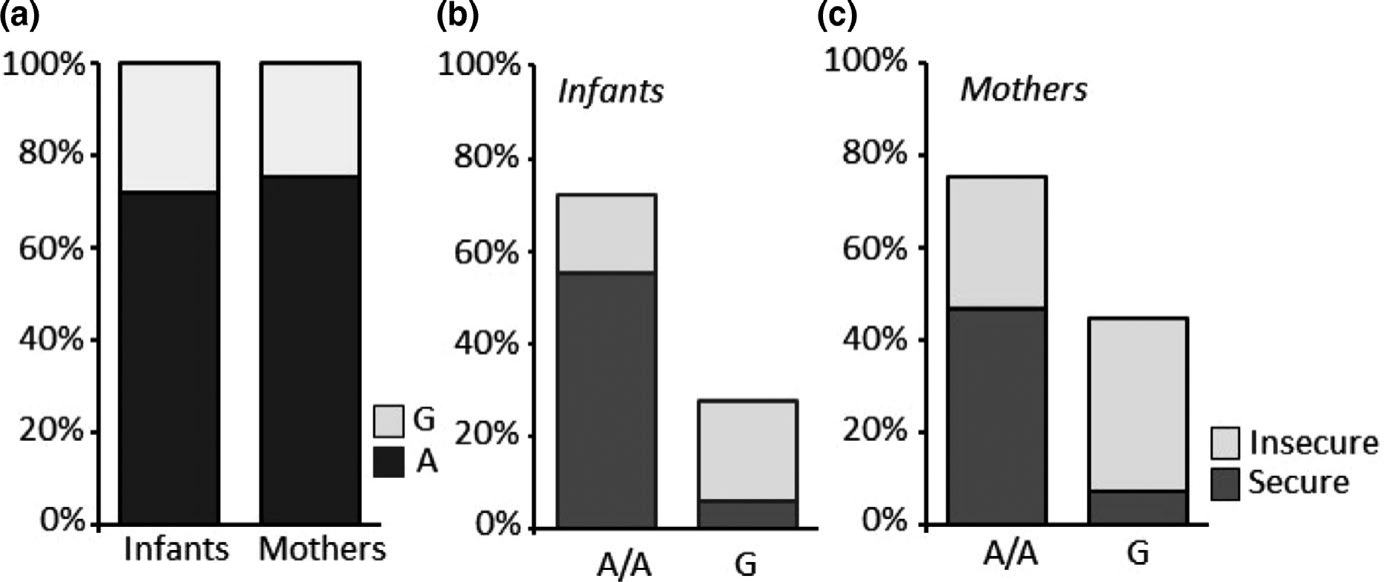
Infant and their mother genotype frequencies were in Hardy–Weinberg equilibrium: mothers 75% A/A and 25% G (A/G, G/G) and infants 72% A/A and 28% G (A/G, G/G) (a). The percentage of A/A homozygous infants (b) and mothers (c) with secure a secure attachment was higher than A/A homozygous infants and mothers with insecure attachment. On the contrary, the percentage of infants and mothers carrying G allele and with insecure attachment was higher than infants and mothers with the same genotype but with secure attachment (b, c)

The frequency distribution of the A118G genotypes in children and their mothers was as follows: children, 72% A/A, 25% A/G, 3% G/G; mothers, 75% A/A, 22% A/G, 3% G/G. Genotypic frequencies of children and their mothers were in Hardy–Weinberg equilibrium (children: chi‐square = 0.09, *df* = 1, *p* = .85; mothers: chi‐square = 1.91, *df* = 1, *p* = .86). As already reported in other studies carried out on European/Caucasian populations (i.e., Sweeney et al., 2017; The International HapMap 3 Consortium, 2010; Troisi et al., 2012), the frequency of the G allele was very low with respect to the A allele (approx. 15% vs. 85%). The G/G and A/G groups were therefore combined in the data analysis in a group of G allele carriers in both mothers and children (children 72% A and 28%G; mothers 75% A and 25% G; Figure 1a), according to the model of Arias, Feinn, and Kranzler (2006) and subsequent studies (Copeland et al., 2011; Troisi et al., 2011, 2012).

Based on the categorical classification (secure vs. insecure) of the ECR, 61% of the children described themselves as having a secure style of attachment. Using the categorical classification (secure vs. insecure) of the ECR, 54% of the youths’ mothers described themselves as having a secure style of attachment. In the control and clinical children samples, the percentages of children describing themselves as having a secure style of attachment were 75% and 51%, respectively. As for the mothers, the percentages of the mothers described themselves as having a secure style of attachment were 66% in the control sample and 45% in the clinical sample (Figure 1a).

**Figure 2:**
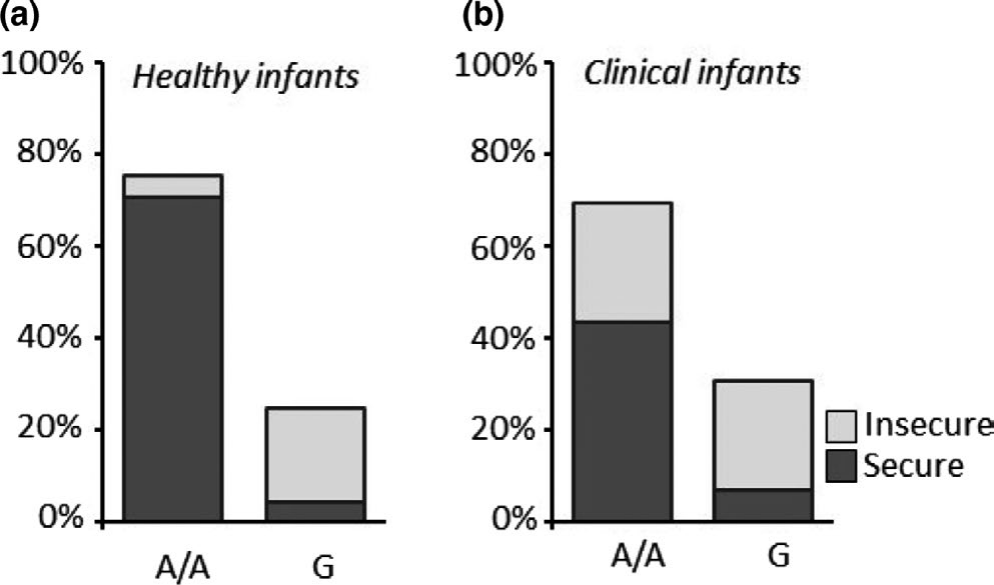
The percentage of healthy A/A homozygous infants with secure attachment was higher than healthy infants with the same genotype but with insecure attachment (a), while the percentage of healthy G‐carrier infants with secure attachment was lower than healthy infants with the same genotype but with insecure attachment (b). The percentage of clinical A/A homozygous infants with insecure attachment increased compared with the healthy infants carrying the same genotype, while no changes in the attachment style distribution were detectable within the G‐carrier groups (a, b)

In the entire children's sample, there was a significant association between the A118G genotype and insecure attachment. The percentage of youths with insecure attachment was significantly higher among carriers of the G allele (chi‐square = 39.17, *df* = 1, phi = −0.51, *p* < .001; Figure 1b). The significant association between the G allele and insecure attachment was confirmed by separate analyses in the subgroups of control children (chi‐square = 36.69, *df* = 1, phi = −0.75, *p* < .001; Figure 2a and 2b) and clinical children (chi‐square = 11.34, *df* = 1, phi = −0.37, *p* = .001; Figure 2b).

**Figure 3:**
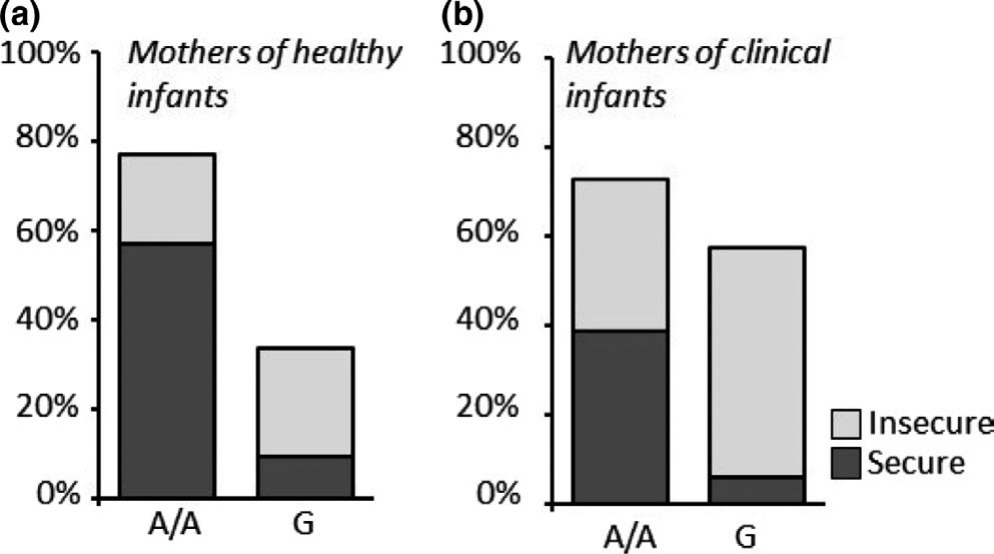
The percentage of healthy infant A/A homozygous mothers with secure attachment was higher than mothers of healthy infants with the same genotype but with insecure attachment (a). On the contrary, the percentage of healthy infant G‐carrier mothers with secure attachment was lower than healthy infant mothers with the same genotype but with insecure attachment (a). The percentage of clinical infant A/A homozygous mothers with insecure attachment increased compared with the healthy infant mothers carrying the same genotype (a, b). In parallel, the percentage of G‐carrier mothers was increased in the clinical context (a, b). Within this last group, the number of mothers with insecure attachment was also increased (b)

In the entire mother sample (including the clinical and the healthy group), there was a significant association between the A118G genotype and insecure attachment. The percentage of mothers with insecure attachment was significantly higher among G allele carriers (chi‐square = 12.04, *df* = 1, phi = −0.28, *p* = .001; Figure 1c). The significant association between the G allele and insecure attachment was confirmed by separate analyses in the subgroups of mothers of control children (chi‐square = 5.96, *df* = 1, phi = −0.30, *p* = .01; Figure 3a) and mothers of clinical children (chi‐square = 6.10, *df* = 1, phi = −0.27, *p* = .01; Figure 3b).

### 3.2 Psychopathological symptoms in G‐carrying mothers and children

**Figure 4:**
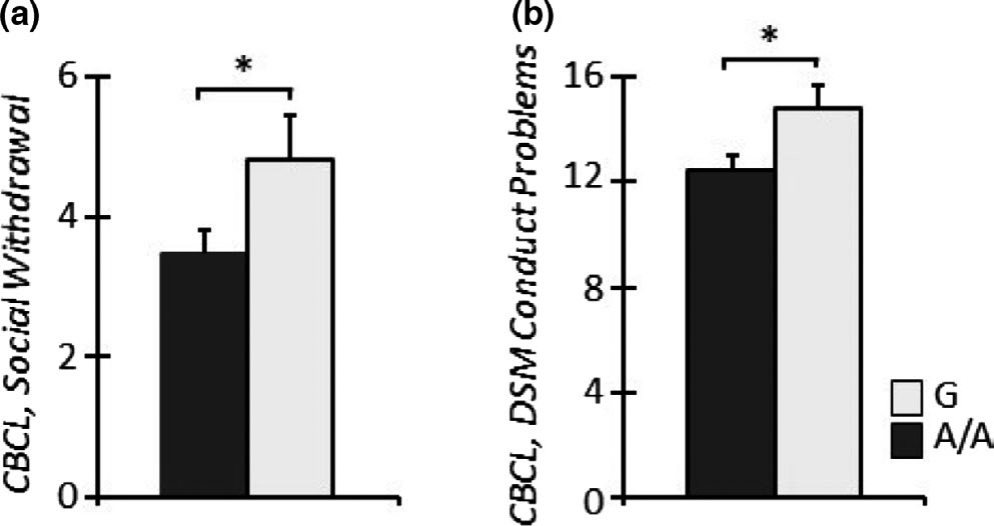
Clinical G‐carrier infants showed higher levels of CBCL‐Social Withdrawal (a) and Conduct Problems (b) compared to clinical A/A homozygous infants. *p < .05

**Figure 5:**
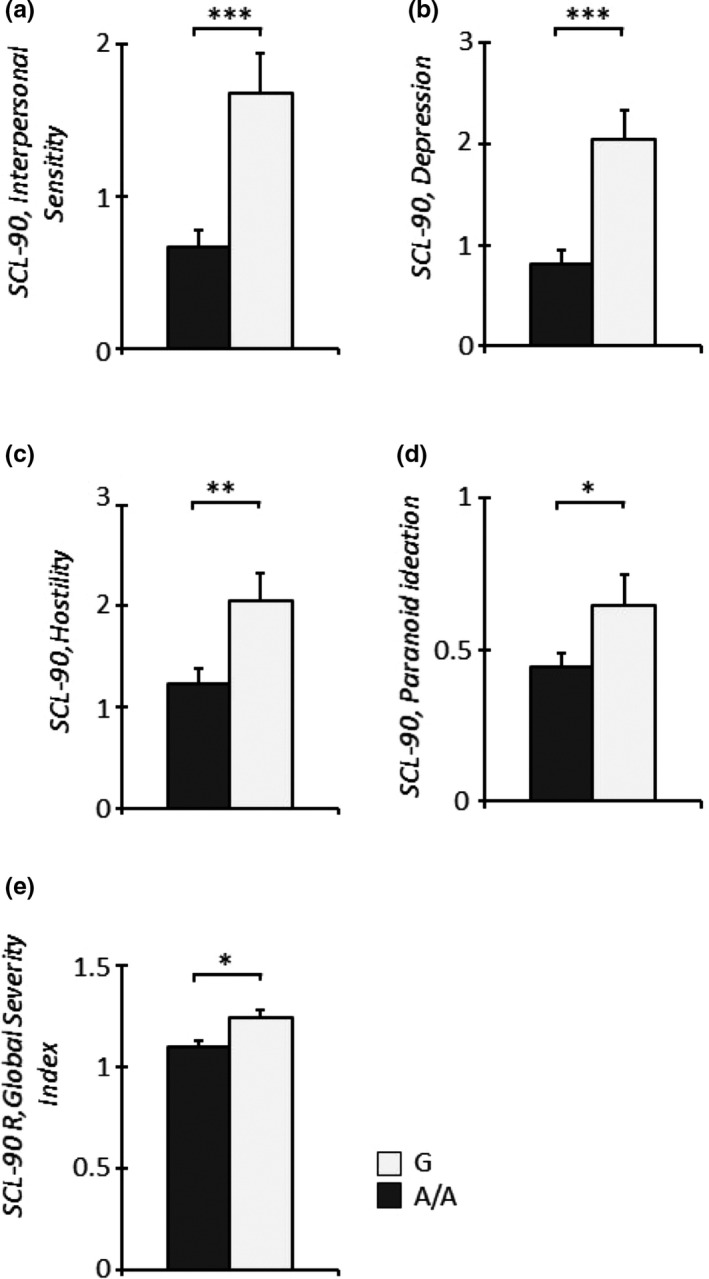
Clinical infant G‐carrier mothers showed higher levels of SCL90/R‐Interpersonal sensitivity (a), Depression (b), Hostility (c), Paranoid ideation (d), and Global Severity Index (e) compared to A/A homozygous mothers of clinical infants. *p < .05, **p < .01, ***p < .001

The impact of the A118G SNP on psychological symptoms of clinical children was evaluated by CBCL. A significant main effect of the *genotype* on the CBCL‐Social withdrawal and CBCL‐Conduct Problem subscales was observed (*Social withdrawal*, *F*_1,83_ = 4.34, *p* = .04; *Conduct Problems,* *F*_1,83_ = 5.11, *p* = .03; Figure 4a,b), with clinical G‐carrying children scoring higher than clinical A/A homozygous children on both subscales. A parallel analysis of the modulating role of the A118G SNP on the psychological status of mothers belonging to the clinical sample, performed by the SCL‐90, revealed a significant main effect of *genotype* on several subscales, *Interpersonal‐Sensitivity (IS), Depression (D), Hostility (H), Paranoid Ideation (PI), and the General Severity Index*, with G‐carrying mothers of clinical children scoring higher than A/A homozygous mothers (*GSI*; *IS*, *F*_1,82_ = 17.74, *p* < .001; *D*, *F*_1,82_ = 20.23, *p* < .001; *H*, *F*_1,82_ = 7.16, *p* = .009; *PI*, *F*_1,82_ = 4.00, *p* = .049; *GSI,* *F*_1,82_ = 5.60, *p* = .02; Figure 5a–e).

### 3.3 Caregiving abilities in G‐carrying mothers

**Figure 6:**
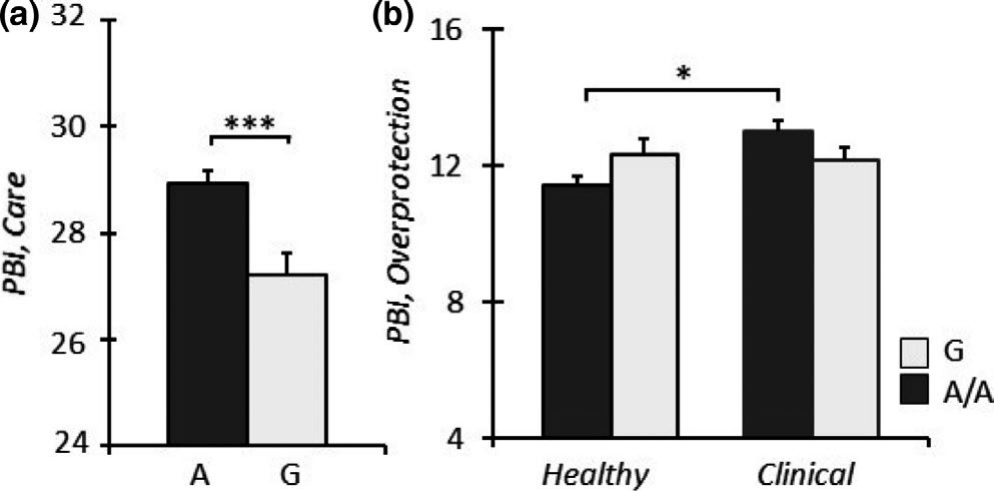
G‐carrier mothers showed lower levels of PBI‐maternal Care (a) compared to A/A homozygous mothers. A/A homozygous mothers of clinical infants showed higher levels of PBI‐maternal overprotection (b) compared to A/A homozygous mothers of healthy infants. *p < .05, and ***p < .001

Finally, we determined the possible association of different OPRM1 genotypes on the amount of mothers’ care. A significant main effect of the *genotype* was observed (*F*_1,145_ = 13.34, *p* < .001), with G‐carrying mothers providing significantly less care than A/A homozygous mothers (Figure 6a). Moreover, a significant interaction effect between *clinical/healthy condition* and *A118G genotype* on the amount of maternal overprotection, measured by the PBI overprotection subscale, was detected (*F*_1,145_ = 4.60, *p* = .034). This indicated that A/A homozygous mothers of clinical children scored higher than A/A homozygous mothers of healthy children (Figure 6b).

## 4 DISCUSSION

The μ‐opioid receptor gene (OPRM1) appears to play a specific role in several aspects of neuronal and psychological activities relative to sensitivity to pain and pleasure, stress response, and social interactions. Its activity is modulated by common sequence variations (see, e.g., Sweeney et al., 2017), and a substantial body of investigation has analyzed the functional A118G SNP. The present study extends these observations to social attachment according to the following hypotheses.

Our first hypothesis was that G allele‐carrying individuals, mothers and children, were more likely to show insecure attachment styles if compared with A/A homozygous individuals. Preliminarily, we verified that 61% (*N* = 91) of the children and 54% (*N* = 81) of the mothers in the total sample described themselves as having a secure style of attachment. These rates are in line with previous literature (van IJzendoorn & Bakermans‐Kranenburg, 2010; Van IJzendoorn MH & Kroonenberg PM., 1988). Interestingly, the percentage of securely attached children and mothers in the clinical sample was, respectively, 51% (*N* = 43) and 45% (*N* = 38), much lower than in the control sample, with 75% (*N* = 48) and 66% (*N* = 42) of youths and mothers, respectively, displaying secure attachment. This observation confirms that children with DMDD experience difficulties in constructing and maintaining positive affective relationships with their caregivers, as suggested in other studies (American Psychiatric Association, 2013; Bell, 1979; Cerniglia, Cimino, Tafà, & Marzilli, 2017; Cicchetti, Toth, & Handley, 2015; Fonagy & Target, 2005; Sameroff, 1975; Uran & Family, 2015). The mothers of these children, on the other hand, showed low rates of secure attachment to their own mothers.

Consistent with our hypothesis, we found that the percentage of mothers and children with insecure attachment was significantly higher among carriers of the G allele, in both the clinical and control groups. This result is in line with the *differential susceptibility model* formulated by Belsky and Pluess (2009) From this standpoint, genetic characteristics act as gain‐of‐function factors rather than as vulnerability features. In fact, in both groups of our sample, but especially in the clinical group, the G allele seemed to act as a magnifying factor, augmenting the frequency of insecure attached individuals. This suggests that due to its predisposing power toward irritability and low tolerance to frustration, OPRM1 amplifies the relational difficulties between youths and caregivers that are already in place as correlates of the severe emotional/behavioral dysregulation of the children. The complex general underpinning mechanism causing this effect remains unclear.

Our second hypothesis was that psychopathological symptoms were higher in G allele‐carrying mothers and children. This idea was confirmed in the clinical sample of G allele‐carrying children, who scored higher than clinical A/A homozygous youths in the subscales of Withdrawal and Conduct Problems. The amplifying effect of the A118G polymorphism can also explain this result, and the higher conduct problems were consistent with the behavioral characteristics of this sample with DMDD children. Less straightforward was the association with higher symptoms of withdrawal. These symptoms, in fact, are not typically noted in children with such a diagnosis (Oyserman, Mowbray, Meares, & Firminger, 2000), whose core features consist of externalizing problems. However, the DSM‐5 does recognize that these children may have difficulties in initiating and sustaining friendships and may avoid most of the activities children usually find enjoyable. In this sense, children with DMDD could be described as withdrawn, and G allele carriers in our sample displayed these characteristics more than their A/A homozygous peers, probably because OPRM1 increases their susceptibility to social rejection. Thus, we speculate that G allele‐carrying children with DMDD could keep themselves particularly distant from social contacts (which are already difficult for youths with this diagnosis) because they were hypersensitive to the interactional problems.

With regard to mothers, a significant main effect of genotype was observed in the clinical group on psychopathological symptoms in several SCL‐90 subscales, such as Interpersonal‐Sensitivity, Depression, Hostility, and Paranoid Ideation, and on the General Severity Index, with G allele‐carrying mothers scoring higher than A/A homozygous mothers on all these subscales. In contrast to their children, these women showed a broad range of symptoms, so that their clinical problems appeared nonspecific. Interestingly, research has found mixed results with regard to the associations between children with DMDD symptoms and their parents’ psychopathology. Some authors have posited that parents of children with DMDD have anxious and depressive symptoms (the latter typically found in mothers (Wiggins, Mitchell, Stringaris, & Leibenluft, 2014). Conversely, other studies have suggested no associations between children with DMDD and parental psychopathology (Axelson et al., 2012). In our study, mothers of children with DMDD, carrying the G allele, showed higher symptoms, so that in those families, we observed youths and mothers (all G carriers) with psychopathological symptoms higher than A/A homozygotes. The biopsychosocial standpoint (Cicchetti, 2007) has documented the fact that the same genetic variation in the OPRM1 could explain some of the phenotypic variance observed in children and in mothers, suggesting that children's and mothers’ emotional/behavioral functioning could have coadapted, giving rise to a familiar problematic configuration with a significant psychopathological risk.

Our third and last hypothesis was that caregiving abilities were lower in G allele‐carrying mothers. A significant main effect of the genotype was observed, with the G carriers providing significantly less care than A/A homozygotes. This finding is in line with previous literature that demonstrated low sensitivity and responsiveness in parents with this genetic variation. It should also be noted that maternal caregiving abilities were assessed through the PBI that was filled out by these mothers’ children. If we consider that these children suffer from a psychiatric condition whose debilitating symptoms are apparently worsened by the A118G variation they carry, we cannot rule out the possibility that their description of maternal care could be distorted.

Altogether, our study offers new insights into the associations between A118G, emotional and behavioral functioning, and attachment style in children and between psychopathology and caregiving ability in mothers.

We acknowledge the following limitations. First, we did not include fathers in our research but left analysis of the possible associations between OPRM1 polymorphism and paternal psychopathology, attachment, and caregiving for a future study. Also, the assessment of mothers’ psychopathological symptomatology was obtained through a self‐report measure (the SCL‐90/R). However, this tool is a reliable one and is widely used for both clinical and nonclinical populations (Prunas et al., 2012). Second, the present research was conducted on a candidate‐gene approach based on previous literature (Rutter, Moffitt, & Caspi, 2006) and on a relatively small sample, similar to most studies of this type. While this limits its statistical power, we choose to focus on a sample with a severe clinical condition (DMDD) that is quite rare (2%–5% of prevalence in clinical samples) and on a very specific genetic variation (A118G of OPRM1), allowing explorative hypotheses on the associations between DMDD and µ‐opioid receptor polymorphism to be evaluated on large samples. Third, consistent with published research, the present analyses compared A/A homozygotes to grouped G/A heterozygotes and G/G homozygotes, given the rarity of the latter and the nature of the nucleotide and amino acid substitution. Additional studies on larger samples are therefore also warranted to overcome this limitation. As for the apparent higher power of a genome‐wide method in the study of psychopathology, it is true that genome‐wide association studies have provided promising results, identifying several genetic markers for individual risk of genetic diseases or conditions, but these generally consist of common variants that explain a small fraction of the overall genetic contribution to such risk, usually not exceeding 20% (Kraft & Aschard, 2015). Fourth, it is highly probable that multiple genes and epigenetic variations act together to form the genetic risk underpinning psychopathology (Cicchetti et al., 2015), thus highlighting the need for thorough investigation to elucidate the complex network of genetic, epigenetic, and environmental factors associated with DMDD, psychopathological functioning, and insecure attachment (Cimino et al., 2018; Cinque et al., 2018). Lastly, since the influence of genetic variations and environmental factors on emotional/behavioral functioning does not remain stable over time (Neale et al., 2010), longitudinal studies should be conducted to confirm these results.

## 5 CONCLUSIONS

The existence of the A118G SNP and several other polymorphisms in the μ‐opioid receptor gene deserves a final consideration. The A118G SNP is characterized by allelic frequencies ranging from 90% to 50% for the more common A allele and from 10% to 50% for the rarer G allele, depending on the populations considered (Sudmant et al., 2015; The International HapMap 3 Consortium, 2010). Interestingly, the prevalence of the latter is low in European populations (around 15%) but is similar (35%–50%) to those found for the A allele in Asiatic populations (Bevilacqua, 2019; Sweeney et al., 2017). At the same time, the A118G SNP is not present in African populations, in which a different SNP is diffused (C17T) although almost absent elsewhere. In both cases, however, both nonsynonymous substitutions are responsible for the existence of fully active and less active μ‐opioid receptor proteins, thus producing alternative phenotypes. The two SNPs have probably arisen recently in human evolution, producing amino acid substitutions in the N‐terminal region of the receptor protein, which thus appears to have a low degree of selection constraint. (In support of this notion is the observation that while synonymous polymorphisms are scattered throughout the receptor, an excess of nonsynonymous changes is present in the N‐terminal region). Across‐species genetic analyses (Barr et al., 2007, 2010; Miller et al., 2004; Schwandt et al., 2011; Vallender, Ruedi‐Bettschen, Miller, & Platt, 2010) and in vitro studies with cloned sequences (Knapman & Connor, 2015b; Sweeney et al., 2017). suggest that two functional distinct types of μ‐opioid receptors have arisen along evolution in various primate species and are being maintained by selective pressure relying on their capability of binding opioid ligands and leading to consequent signal transduction. One hypothesis on the effects of these polymorphisms is that they produce individuals who are high‐reactive and low‐reactive to stress, via the hypothalamic–pituitary–adrenal axis (Ducat et al., 2013; Hernandez‐Avila et al., 2007; Wand et al., 2002), and, consequently, that both phenotypes are adaptive, probably depending on different environmental conditions frequently observed in our species and shared with other primate species. Understanding the nature of these conditions would be important to unveiling the selective pressure acting on the opioid system and obtaining a conceptual framework for the physical/psychological traits affected by this system and their approach when clinically relevant.

## AUTHOR CONTRIBUTIONS

SC designed the study and contributed to write the manuscript and to collect and interpret the data. VC contributed to design the study and to write the manuscript. She also analyzed and interpreted the data and prepared the figures. LC contributed to design the study, to write the manuscript, and to interpret the data. SB performed the biological assessment and assisted in writing the manuscript. AB contributed to design the study and to write the manuscript. RT assisted in designing the study and interpreting the data; she also supervised the whole research. All authors have read and approved the manuscript.

## COMPETING INTERESTS

The authors declare that they have no conflict of interest.

## Data Availability

With this manuscript, we provide the study protocol but no patient data.
